# BURDEN OF FALL-RISK INCREASING DRUGS IN OLDER ADULTS PRESENTING WITH FALLS TO THE EMERGENCY ROOM

**DOI:** 10.1093/geroni/igac059.3099

**Published:** 2022-12-20

**Authors:** Martin Casey, Joshua Niznik, Greta Anton, Michelle Meyer, Casey Kelley, Jan Busby-Whitehead, Kathleen Davenport, Ellen Roberts

**Affiliations:** University of North Carolina at Chapel Hill, Chapel Hill, North Carolina, United States; University of North Carolina at Chapel Hill, Chapel Hill, North Carolina, United States; University of North Carolina Medical Center, Chapel Hill, North Carolina, United States; University of North Carolina at Chapel Hill, Chapel Hill, North Carolina, United States; Unicersity of North Carolina at Chapel Hill, Chapel Hill, North Carolina, United States; University of North Carolina at Chapel Hill, Chapel Hill, North Carolina, United States; Univerisity of North Carolina at Chapel Hill, Chapel Hill, North Carolina, United States; University of North Carolina at Chapel Hill, Chapel Hill, North Carolina, United States

## Abstract

Prescribing of fall-risk increasing drugs (FRIDs) may be an important driver of falls in older adults. Understanding the types and frequencies of FRIDs prescribed to older adults presenting with falls to an emergency department (ED) may help identify opportunities for deprescribing. We performed a cross sectional analysis of data collected from a pharmacist-led fall-prevention program focused on older adults presenting with a fall to an academic ED in the southeastern United States between August 2020 – December 2021. ED pharmacists identified older adults (≥65 years old) presenting with a chief complaint of ‘fall’ and then performed a medication reconciliations to verify and obtain data on outpatient prescription drug use. FRIDs were identified in accord with the 2019 American Geriatrics Society Beers Criteria and the Centers for Disease Control and Prevention Stopping Elderly Accidents, Deaths, & Injuries (STEADI-Rx) list. The ED pharmacists performed medication reconciliations on 424 unique older adults presenting with a fall. The cohort had a mean age of 81.3 years and were mostly female (63.3%) and white (84.9%). Prescription use of FRIDs were identified in 45.8% (194/424) of older adults presenting with a fall. An estimated 25.5% (108/424) of the subjects were prescribed 2 or more FRIDs. The most common FRIDs identified were antidepressants (25.9%), anticonvulsants (18.6%), opioids (12.7%), benzodiazepines (8.5%), and antipyschotics (3.1%). . Prescription use of FRIDs, including use of 2 or more FRIDs, was common in older adults presenting with a fall to the ED.

